# Huoxiang Zhengqi alleviates azoxymethane/dextran sulfate sodium-induced colitis-associated cancer by regulating Nrf2/NF-κB/NLRP3 signaling

**DOI:** 10.3389/fphar.2022.1002269

**Published:** 2022-10-21

**Authors:** Mingyuan Dong, Honghan Liu, Tianjiao Cao, Lanzhou Li, Zhen Sun, Ye Qiu, Di Wang

**Affiliations:** ^1^ Department of Pharmacy, Changchun University of Chinese Medicine, Changchun, China; ^2^ School of Life Sciences, Jilin University, Changchun, China; ^3^ Department of Integration of Chinese and Western Medicine, School of Basic Medical Sciences, Peking University, Beijing, China; ^4^ Engineering Research Center of Chinese Ministry of Education ford Eible and Medicinal Fungi, Jilin Agricultural University, Changchun, China

**Keywords:** Huoxiang Zhengqi, colitis-associated cancer, intestinal microbiota, metabolomics, Nrf2/NF-κB/NLRP3

## Abstract

Colitis-associated cancer (CAC) is a subtype of inflammatory bowel disease (IBD)-associated colorectal cancer. Huoxiang Zhengqi (HXZQ) is a classical Chinese herbal medicine and has been used to treat intestinal disorders, however, anti-CAC effects and underlying mechanisms of HXZQ have not been reported. An azoxymethane/dextran sulfate sodium-induced CAC mice model was used to investigate the anti-CAC effect of HXZQ. HXZQ significantly reduced colonic inflammation, suppressed the size and number of tumors, and reduced the levels of pro-inflammatory cytokines (interleukin [IL]-1α, IL-1β, IL-6, IL-17A, IL-21, IL-23, granulocyte macrophage-colony stimulating factor, and tumor necrosis factor-α) and oxidative stress markers (reactive oxygen species and malondialdehyde), and increased the levels of anti-inflammatory cytokines (IL-10 and IL-27) in CAC mice. Intestinal microbiota and serum metabolomics analyses indicated that HXZQ altered the gut microbial composition and the abundance of 29 serum metabolites in CAC mice. Additionally, HXZQ activated the nuclear factor-erythroid factor 2-related factor 2 (Nrf2) signaling pathway and increased the levels of antioxidants such as catalase (CAT), heme oxygenase-1 (HO-1), NAD(P)H quinone oxidoreductases-1 (NQO-1), and superoxide dismutase-1 (SOD-1). HXZQ inhibited the activation of the nuclear factor kappa-B (NF-κB) signaling pathway and decreased the expression of NLR family pyrin domain containing 3 (NLRP3) by inhibiting the phosphorylation of inhibitor of nuclear factor kappa-B (IκB), inhibitor of nuclear factor kappa-B kinase (IKK), and NF-κB. In conclusion, HXZQ alleviated CAC in mice by modulating the intestinal microbiota and metabolism, activating Nrf2-mediated antioxidant response, and inhibiting NF-κB-mediated NLRP3 inflammasome activation against inflammation. The present data provide a reference for the use of HXZQ as a therapeutic or combination agent for clinical CAC treatment.

## 1 Introduction

Colitis-associated cancer (CAC) is a subtype of inflammatory bowel disease (IBD)-associated colorectal cancer. IBD includes Crohn’s disease (CD) and ulcerative colitis (UC) (Terzić et al., 2010). Inflammatory states of patients with IBD tend to induce mutations, epigenetic alterations, and genomic instability, leading to CAC (Kusunoki et al., 2016). No less than 20% of patients with IBD develop CAC within 30 years of onset, and 50% of them die of the disease (Terzić et al., 2010). Surgery is the gold-standard therapeutic regimen for patients with CAC (Kusunoki et al., 2016); however, surgical excision leads to a decrease in the intestinal absorption area (Ananthakrishnan et al., 2017). According to current research, the application of chemoprophylaxis, such as oxaliplatin and fluorouracil (Xiao J. et al., 2016; Luo et al., 2020), reduces the risk of CAC by reducing inflammation in IBD (Kusunoki et al., 2016). Unfortunately, these drugs have common side effects, such as nausea, diarrhea, loss of appetite (Network, 2021b; a), and dysfunction in digestion and absorption (McQuade et al., 2016; McQuade et al., 2018).

The transition from chronic inflammation to carcinogenicity can be driven by oxidative stress and inflammatory cytokines (Fantini and Guadagni, 2021). According to a previous study, tumor necrosis factor-alpha (TNF-α) and interleukin-6 (IL-6) can promote CAC development (Yao et al., 2019). Furthermore, activated nuclear factor-erythroid factor 2-related factor 2 (Nrf2), the primary regulator of the antioxidant response (Bellezza et al., 2018), can inhibit the assembly of the NLR family pyrin domain containing 3 (NLRP3) inflammasome, thereby preventing dextran sulfate sodium (DSS)-induced colitis (Ahmed et al., 2017). Atractylenolide III, the main component of *Atractylodes*, regulates oxidative stress through the Nrf2 pathway and alleviates 2,4,6-trinitrobenzenesulfonic acid-induced acute colitis by influencing the composition of intestinal microbiota (Ren et al., 2021). *Pingkui* enema may reduce IL-8 and TNF-α levels, increase IL-13 levels in UC rat serum, and increase intestinal mucosal bifidobacterial adhesion and adhesion receptor levels to alleviate symptoms (Han D. et al., 2019).

Furthermore, in addition to inflammatory cytokines promoting cancer transitions, during the progression from IBD to CAC, inflammation caused by oxidative stress exacerbates the inflammatory response and increases reactive oxygen species (ROS) production, which affects the homeostasis of the intestinal microbiota (Biasi et al., 2013). ROS can lead to DNA damage and mutations, inflammation and inflammation-derived endogenous DNA damage agents can accelerate the development of CAC, while ROS suppressed or cleared was effective in reducing DNA damage and delaying intestinal tumors (Grivennikov, 2013). In chronic inflammatory conditions, while how specific changes in the intestinal microbiota promote cancer are unclear, there is already evidence linking the intestinal microbiota to inflammation and CAC, such as *Bacteroides fragilis* (BF) that does not express BF enterotoxin to prevent DSS-induced colitis and prevent the formation of polyps in the model of CAC(Fantini and Guadagni, 2021).

Huoxiang Zhengqi (HXZQ) is a classic Chinese herbal medicine (Supplementary Table S1) that was recorded in the *Prescriptions of Peaceful Benevolent Dispensary* for approximately 900 years and has been used to treat intestinal disorders (Zhao et al., 2019). Recent research has shown that HXZQ improves the adult intestinal microbiota and significantly downregulates IL-6, IL-1β, and TNF-α expression in the plasma of antibiotic cocktail-induced gut dysbiosis mice(Gao et al., 2022). Norfloxacin combined with HXZQ in the form of pills effectively reduces symptoms and alleviates pain in acute gastroenteritis (Xuan, 2013). However, to date, the anti-CAC effects of HXZQ and the underlying mechanisms have not been reported.

The azoxymethane (AOM)/DSS-induced CAC mouse model mimics the pathology of CAC in humans and has been used for screening agents with anti-CAC properties and revealing the underlying mechanisms (Parang et al., 2016). In the present study, HXZQ showed anti-CAC effects regulating Nrf2/NF-κB/NLRP3 signaling in AOM/DSS-induced CAC mice. This study provides a reference for the application and development of HXZQ in the clinical treatment of CAC.

## 2 Materials and methods

### 2.1 Animal experimental protocol

Thirty-two 6-week-old male C57BL/6 mice (SCXK(Liao)2020–0,001, Liaoning Changsheng Biotechnology Co., Ltd. Benxi, China) were housed under specific pathogen-free conditions at the appropriate temperature (22 ± 2°C) and humidity (50% ± 10%) and kept on a 12/12 h light/dark cycle. The mice had unrestricted access to food and water. The experimental protocol complied with the ARRIVE guidelines and was approved by the Institution Animal Ethics Committee of Jilin University (SY202104007).

**FIGURE 1:**
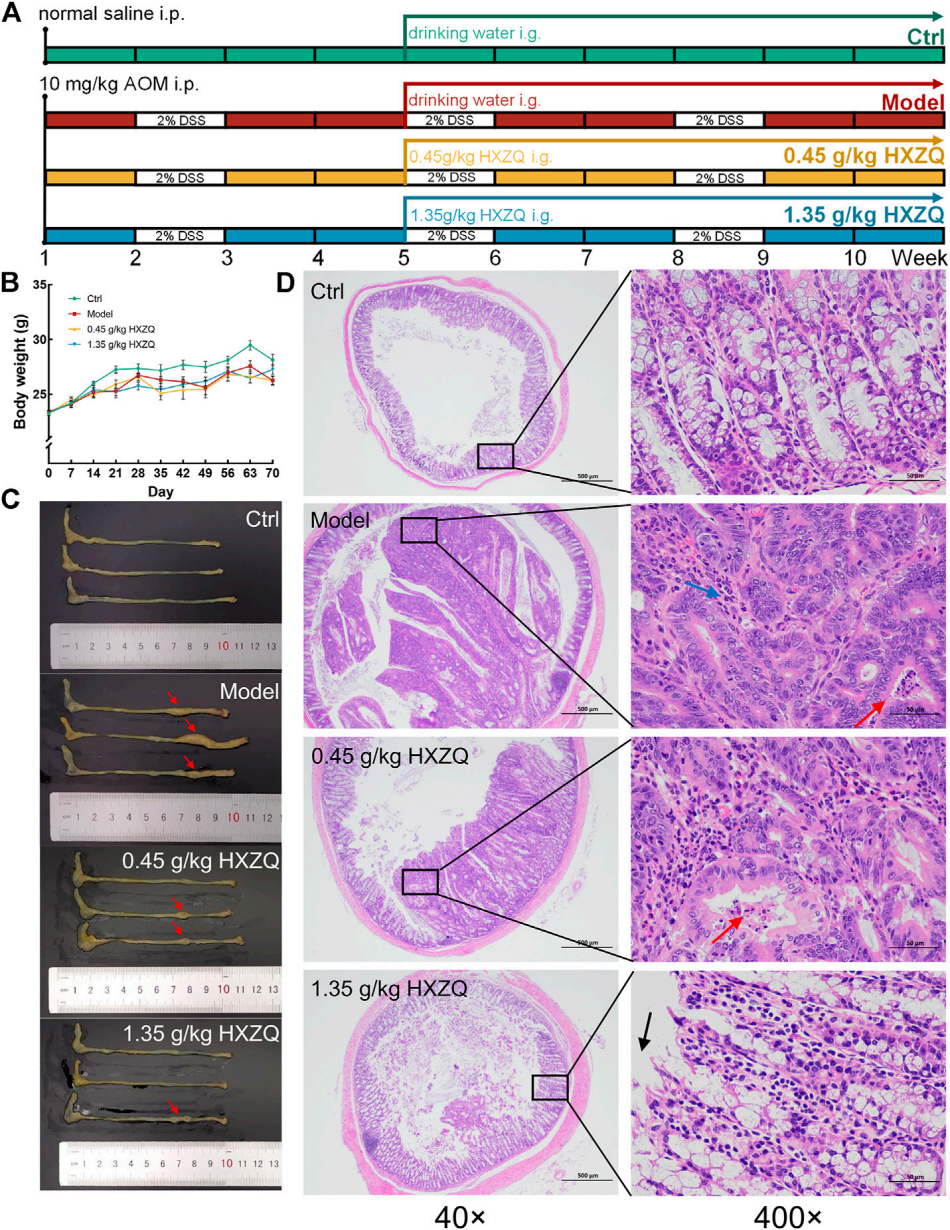
The anti-CAC effect of HXZQ on AOM/DSS mice. **(A)** Schematic overview of the experimental protocol. **(B)** Body weight was monitored every week during the entire experimental period (n = 8). **(C)** HXZQ suppressed the tumor growth in AOM/DSS mice (*n* = 3). **(D)** Histopathological observation of colorectal tumor in AOM/DSS mice (40 × scale bar: 500 μm; 400 × scale bar: 50 μm) (*n* = 3).

Twenty-four mice were intraperitoneally injected with 10 mg/kg of AOM (#A5486, Sigma-Aldrich, St Louis, MO, United States) on the first day, and their drinking water was changed to 2% DSS (#S14049, Shanghaiyuanye Bio-Technology Co., Ltd., Shanghai, China) at the second, fifth, and eighth week. From the fifth week and beyond, the mice were randomly divided into three groups and orally received normal saline (*n* = 8) (serving as the model group) or 0.45 or 1.35 g/kg HXZQ (#200301, XiuZheng Pharmaceutical Group Co., Ltd., Changchun, China) (serving as the HXZQ-treated groups) daily for 6 weeks. Another eight mice were intraperitoneally injected with normal saline on the first day, received normal drinking water for the entire experimental period, and received normal saline orally daily from the fifth to 10^th^ week (serving as the control [Ctrl] group) (Figure 1A). Six hours after the last administration, the mice were bled *via* the tail vein, euthanized by carbon dioxide asphyxiation, and dissected to collect the colon, liver, spleen, kidney, and cecum contents. The size and number of tumors were examined and recorded by two independent observers.

### 2.2 Histopathological examination

The fixed colon, liver, spleen, and kidney tissues were paraffin-embedded, sectioned, and dewaxed in xylene for 40 min, anhydrous ethanol for 10 min, and 75% ethanol for 5 min, and then washed with running water. The sections were stained with hematoxylin and eosin (H&E) and treated sequentially with ethanol and xylene for dehydration. The sealed sections were observed and analyzed using an ECLIPSE E100 upright optical microscope (Nikon, Tokyo, Japan) (Liu et al., 2021).

### 2.3 Immunofluorescence analysis

Paraffin slides of colorectal tumors were subjected to antigen retrieval after de-paraffinization and blocked with 3% bovine serum albumin. The slides were incubated overnight at 4°C with primary antibody for nuclear factor kappa-B factor kappa-B (NF-κB) p65 (SupplementaryTable S2) in a wet box. The target tissue was then covered with fluorescent-labeled secondary antibody (Supplementary Table S2) and incubated for 50 min in the dark at room temperature. The slides were incubated with 4′6-diamidino-2-phenylindole (DAPI) (#G1012, Wuhan servicebio technology Co., Ltd., Wuhan, China) solution for 10 min at room temperature and kept in the dark. Spontaneous fluorescence quenching reagent was added to the slides and incubated for 5 min. Proteins were detected using Ortho-Fluorescent Microscopy (Nikon, Tokyo, Japan).

### 2.4 Intestinal microbiota analysis

The cecum contents obtained from Ctrl, Model, and HXZQ-treated mice (*n* = 4) were used for 16S rRNA analysis of the gut microbiota. Nucleic acids were extracted from the contents of each cecum, and PCR amplification of the V3-V4 region of the bacterial 16S rRNA gene was performed. PCR products were quantified and 2 × 250 bp double-end sequencing was performed. Sequencing was performed at Shanghai Personal Biotechnology Co. Ltd. (Shanghai, China).

The 16S rRNA sequencing results were clustered into amplicon sequence variants (ASVs) using DADA2 with 100% similarity. Based on the ASV abundance data, a flower plot was generated, and alpha diversity indices and the weighted UniFrac distance matrix were calculated. Abundance data from each group of microbiota were used to generate a species composition heatmap and for LDA effect size (LEfse) analysis. Microbial functions were predicted using Phylogenetic Investigation of Communities by Reconstruction of Unobserved States. The sequences of bacteria were uploaded to the NCBI Sequence Read Archive with accession number PRJNA860221 (https:// www.ncbi.nlm.nih.gov/sra/PRJNA860221). These analyses were performed as previously described (Ling et al., 2014; Jiang et al., 2021).

### 2.5 Metabolomics analysis

Serum samples were thawed slowly at 4°C, and 100 µL of the sample was added to 400 μL of a pre-cooled methanol/acetonitrile solution (1:1, v/v) for vortex mixing. The supernatant obtained by centrifugation of the mixture was vacuum dried to a solid state. Before the analysis, the samples were dissolved in an aqueous solution of acetonitrile (acetonitrile: water = 1:1, v/v). Samples were separated on an ultra-high-performance liquid chromatography system (1,290 Infinity LC, Agilent Technologies, Palo Alto, Calif. United States) equipped with a ACQUITY UPLC BEH Amide 1.7 μm, 2.1 mm × 100 mm column (Waters, Milford, MA, United States) and were analyzed using a quadrupole-time of flight mass spectrometer (AB Sciex TripleTOF 6,600, Framingham, MA, United States). The separation conditions were identical to those reported in previous studies (Qu et al., 2021).

The processed data were subjected to orthogonal partial least squares discriminant analysis (OPLS-DA), and score plots were plotted. The abundance of signature metabolites screened with OPLS-DA VIP >1 and *p*-value <0.05 as filtering criteria were used to draw heatmaps and correlation plots. The abundance data of the microbiota and metabolites with significant changes were jointly analyzed to obtain association heatmaps. These analyses were performed as described in our previous study (Jiang et al., 2021).

### 2.6 Biochemical analyses

The collected tumor tissues were homogenized separately in phosphate buffer solution (PBS). Protein concentrations were determined using a Pierce™ BCA Protein Assay Kit (#23227, Thermo Fisher Scientific, Waltham, MA, United States). IL-1α, IL-1β, IL-6, IL-17A, IL-23, IL-27 and TNF-α in tumor tissues were detected using the LEGENDplex™ Mouse Inflammation Panel (13-plex) with a V-bottom plate (#740446, Biolegend, San Diego, CA, United States). Enzyme-linked immunosorbent assay (ELISA) kits (Jiangsu MEIMIAN, Jiangsu, China) were used to measure IL-10 (#MM-0176M1), IL-21 (#MM-0688M1), granulocyte-macrophage colony-stimulating factor (GM-CSF) (#MM-0185M1), malondialdehyde (MDA) (#MM-0897M1), and ROS (#MM-43700M1) levels in tumors.

### 2.7 Western blotting

Tumor tissues were lysed at low temperatures in Radioimmunoprecipitation assay (RIPA) Buffer (#20–188, Merck Millipore, MA, United States) supplemented with 1% Protease and Phosphatase Inhibitor Cocktail (#P002, NCM Biotechnology Co., Ltd. Suzhou, China). Cytoplasmic and nuclear proteins in tumors were isolated by NE-PER Nuclear and Cytoplasmic Extraction Kit (#78833, Thermo Fisher Scientific, Waltham, MA, United States). Total protein in each sample was measured using the Pierce™ BCA Protein Assay Kit (#23227, Thermo Fisher Scientific, Waltham, MA, United States). The protein samples were separated using 12% sodium dodecyl sulfate-polyacrylamide gel electrophoresis. The proteins were transferred to PVDF membranes, and the membranes were blocked with western fast blocking solution (#GF 1815, Genefist, Oxfordshire, United Kingdom) for 10 min at 25°C. The membranes were incubated with primary antibodies (Supplementary Table S2) for 12 h at 4°C and then incubated with the appropriate secondary antibodies (Supplementary Table S2) for 4 h at 4°C. The Ultra-High Sensitivity ECL kit (#GK10006, GLPBIO, Montclair, CA, United States) was used to develop the signal, and the intensity of protein expression was measured using a Tanon 5,200 imaging system (Tanon Science & Technology Co., Ltd., Shanghai, China). Photographic results were quantitatively analyzed using the ImageJ v1.8.0 (National Institutes of Health, Bethesda, MD, United States).

### 2.8 Statistical analysis

All values are presented as mean ± standard error of the mean (S.E.M.) Biochemical indices were compared between Ctrl and Model using Student’s t-test. Comparisons among Model, 0.45 g/kg HXZQ, and 1.35 g/kg HXZQ were performed with one-way analysis of variance (ANOVA) followed by a post hoc multiple comparisons (Dunnett) test using BONC DSS Statistics 25 software (Business-intelligence of Oriental Nations Co., Ltd. Beijing, China). A *p*-value less than 0.05 was considered statistically significant.

## 3 Results

### 3.1 HXZQ significantly inhibits tumor growth in CAC mice

HXZQ remarkably reduced the number and size of colonic tumors in CAC mice without influencing their body weight (*p* < 0.05) (Figures 1B, C and Supplementary Figure S1). Compared with the Ctrl group, the Model group showed heterogeneous nuclei, high nucleoplasmic ratios and inconspicuous nucleoli in colonic cancer cells, a small amount of necrotic cell debris in the cancerous tissue, and reduced granulocyte infiltration in the interstitium in the colorectum; all these effects were reversed after HXZQ administration (Figure 1D). HXZQ failed to influence other organ structures, including the liver, spleen, and kidneys, of the CAC mice (Supplementary Figure S2).

### 3.2 HXZQ regulates the intestinal microbiota in CAC mice

**FIGURE 2:**
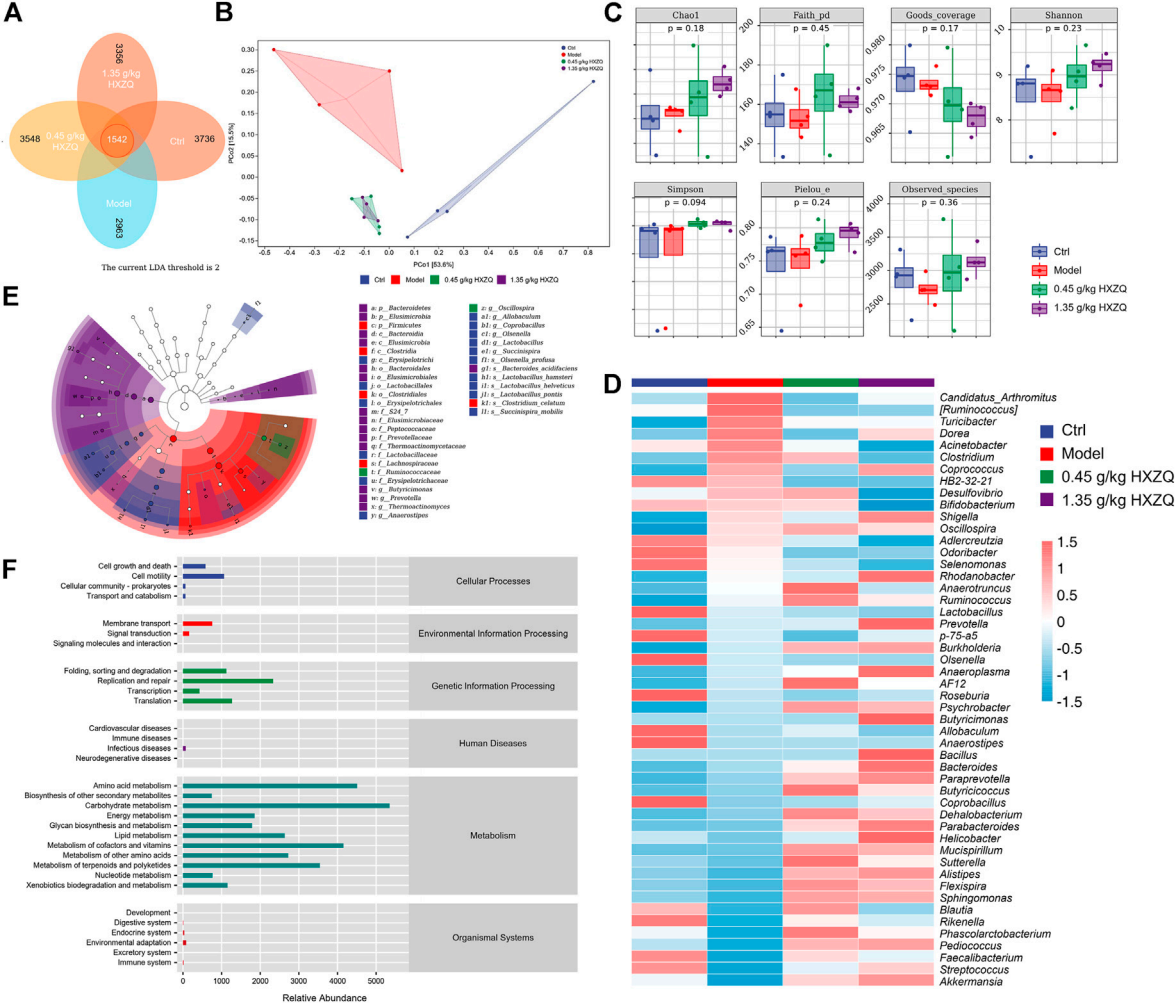
The effect of HXZQ on the intestinal microbiota of AOM/DSS mice (*n* = 4) **(A)** Venn diagram. **(B)** PCoA of weighted UniFrac distance from beta diversity analysis. **(C)** Grouping box plot of alpha diversity index includes Chao1, Faith_pd, Goods_coverage, Shannon, Simpson, Pielou_e, and Observed_species of intestinal microbiota. **(D)** Heatmap of the top 50 dominant species composition at the genus level. **(E)** LEfSe analysis of the dominant biomarker taxa among the four groups. The threshold of the logarithmic score of LDA analysis was 2.0. **(F)** Predicted abundance graph of KEGG metabolic pathways.

Among the 13,603 detected ASVs, 1,542 were shared by the four groups. The number of ASVs in the Ctrl, Model, 0.45 g/kg HXZQ-treated, and 1.35 g/kg HXZQ-treated groups was 3,736, 2,963, 3,548, and 3,356, respectively (Figure 2A). According to the beta diversity obtained by principal coordinate analysis (PCoA) assessment, separation of microbial composition was observed between the model group and HXZQ-treated groups (Figure 2B). HXZQ did not influence the alpha diversity index (Figure 2C). According to the heatmap, AOM/DSS resulted in increased levels of *Candidatus_Arthromitus*, *Turicibacter*, *Dorea*, *Acinetobacter*, *Clostridium,* and *Desulfovibrio*, which were suppressed by HXZQ. Compared to the model group, HXZQ resulted in an increased abundance of 25 genera (Figure 2D and Supplementary Table S3).

HXZQ caused structural changes in the microbiota of CAC mice. In contrast, there was no significant difference in microbiota between the 0.45 and 1.35 g/kg HXZQ-treated groups (Figure 2B). Linear discriminant analysis effect size (LEfSe) analysis used to identify robust biomarkers between subgroups showed that a total of 17 taxa were tagged as biomarkers in the HXZQ-treated group, including Bacteroidia, S24-7, *Butyricimonas*, *Bacteroides_acidifaciens*, *Prevotella*, Ruminococcaceae, and *Oscillospira*. The model group had five prominent taxa: Firmicutes and its subordinate taxa, including Clostridia, Clostridiales, Lachnospiraceae, and *Clostridium_celatum* (*p* < 0.05, LDA >2) (Figure 2E). The abundance values of metabolic pathways were obtained from the KEGG metabolic pathway database. The top six predicted pathways were carbohydrate metabolism (abundance value: 5,349.51), amino acid metabolism (4,509.27), metabolism of cofactors and vitamins (4,155.2), metabolism of terpenoids and polyketides (3,544.08), metabolism of other amino acids (2,723.91), and lipid metabolism (2,635.33) (Figure 2F). Comparative analysis of the metabolic pathways revealed that the phosphotransferase system (PTS) was significantly inhibited by HXZQ (*p* < 0.001) (Supplementary Figure S3).

### 3.3 HXZQ alters the metabolism in CAC mice

**FIGURE 3:**
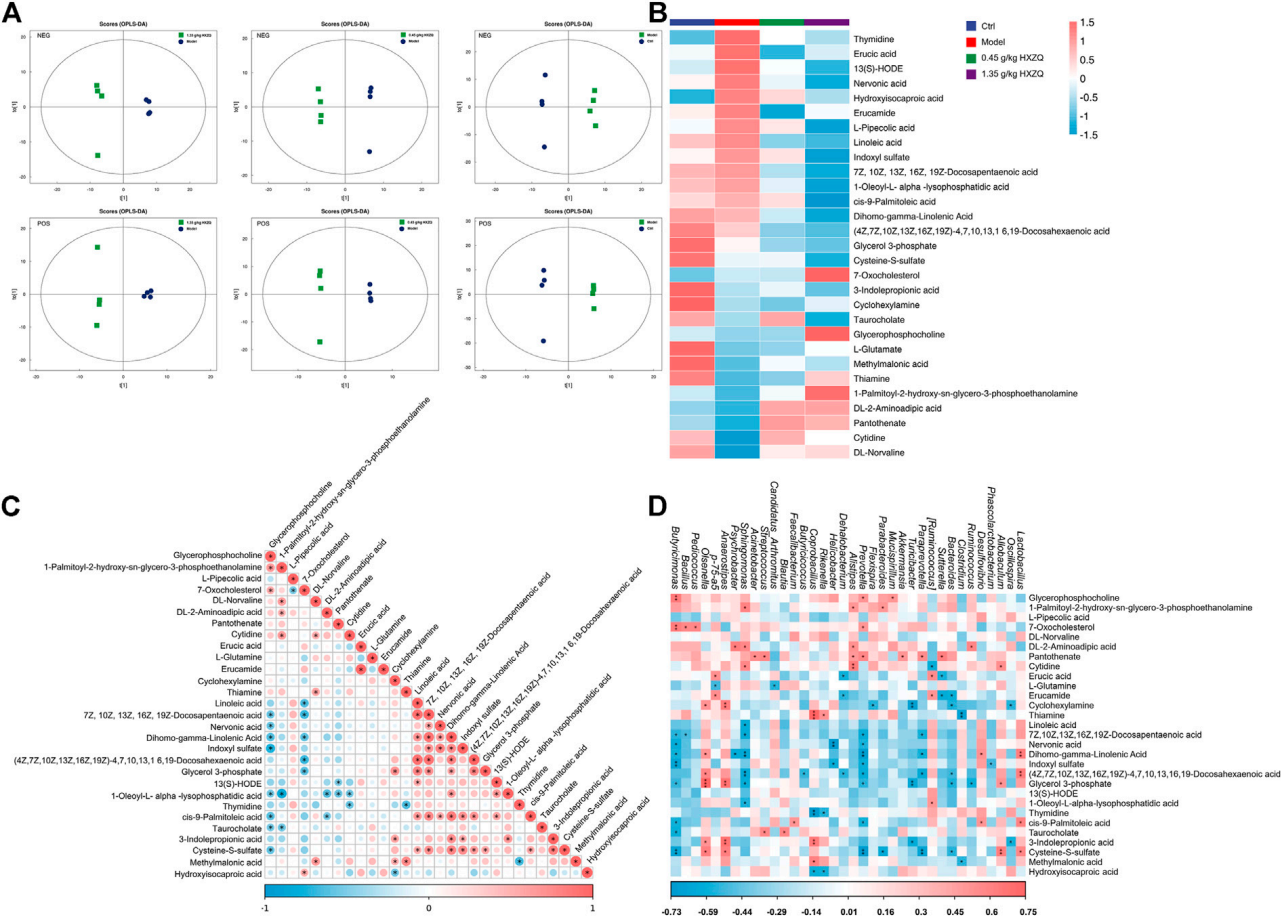
HXZQ regulated serum metabolite levels in AOM/DSS mice **(A)** OPLS-DA score plot. **(B)** Heatmap of 29 significantly altered metabolites in HXZQ-treated AOM/DSS mice. **(C)** The associated heatmap of altered metabolites. **(D)** The associated heatmap of significantly altered metabolites and microbiota species.

The score plots of OPLS-DA showed significant differences in metabolite levels between the model and HXZQ-treated mice (Figure 3A). The levels of 16 metabolites, including linoleic acid, dihomo-gamma-linolenic acid, indoxyl sulfate, and thymidine, were downregulated in the serum of the HXZQ-treated mice (Figure 3B and Supplementary Table S4). HXZQ treatment resulted in upregulation of the levels of 10 metabolites, including L-Glutamine, glycerophosphocholine, and thiamine (Figure 3B and Supplementary Table S4). According to the correlation analysis among metabolites, the common colorectal cancer marker metabolite indoxyl sulfate showed a positive correlation with 3-indolepropionic acid, cis-9-palmitoleic acid, and cysteine-S-sulfate and a negative correlation with glycerophosphocholine (*p* < 0.05) (Figure 3C).

Serum metabolite concentrations were significantly correlated with changes in colony abundance. Indoxyl sulfate was negatively correlated with *Phascolarctobacterium*, *Prevotella, Helicobacter*, and *Butyricimonas* (*p* < 0.05) (Figure 3D). L-Glutamine showed a significant negative correlation with *Candidatus_Arthromitus* and *p-75-a5* (*p* < 0.05) (Figure 3D).

### 3.4 HXZQ suppressed the inflammatory and oxidative response in CAC mice

**FIGURE 4:**
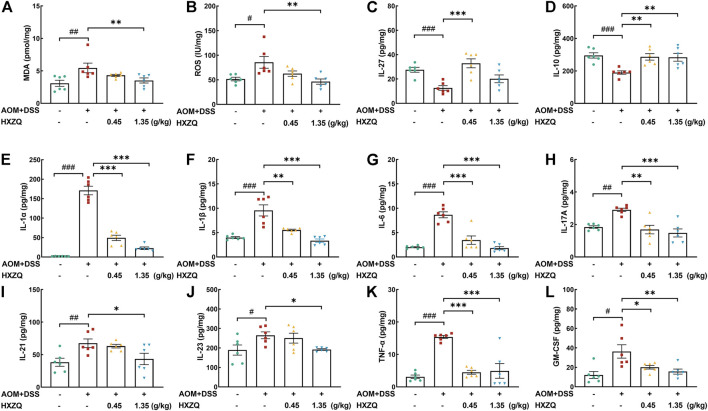
The effects of HXZQ on colorectum cytokines of AOM/DSS mice, including oxidative stress factors **(A)** MDA and **(B)** ROS, anti-inflammatory cytokines, **(C)** IL-27 and **(D)** IL-10, and pro-inflammatory factors, **(E)** IL-1α, **(F)** IL-1β, **(G)** IL-6, **(H)** IL-17A, **(I)** IL-21, **(J)** IL-23, **(K)** TNF-α, **(L)** GM-CSF. Data are presented as the means ± S.E.M. and biochemical indices were compared between Ctrl and Model using Student’s t-test. Comparisons between Model, 0.45 g/kg HXZQ, and 1.35 g/kg HXZQ were performed with one-way ANOVA followed by a post hoc multiple comparisons (Dunnett) test (*n* = 6). ^#^ *p* < 0.05, ^##^ *p* < 0.01, and ^###^ *p* < 0.001 vs. Ctrl group; *p < 0.05, **p < 0.01, and ***p < 0.001 vs. model group.

Inflammation severity tended to correlate positively with CAC development(Hirano et al., 2020). HXZQ dose-dependently reduced the levels of oxidative stress factors including MDA (35% at 1.35 g/kg) (*p* < 0.01) (Figure 4A) and ROS (45% at 1.35 g/kg) (*p* < 0.01) (Figure 4B), and reduced the levels of pro-inflammatory factors including IL-1α (>71%) (*p* < 0.001) (Figure 4E), IL-1β (>42%) (*p* < 0.01) (Figure 4F), IL-6 (>60%) (*p* < 0.001) (Figure 4G), IL-17A (>41%) (*p* < 0.01) (Figure 4H), IL-21 (35% at 1.35 g/kg) (*p* < 0.05) (Figure 4I), IL-23 (27% at 1.35 g/kg) (*p* < 0.05) (Figure 4J), TNF-α (>68%) (*p* < 0.001) (Figure 4K), GM-CSF (>40%) (*p* < 0.05) (Figure 4L). Correspondingly, HXZQ significantly upregulated the levels of anti-inflammatory cytokines including IL-27 (160% at 0.45 g/kg) (*p* < 0.001) (Figure 4C) and IL-10 (>49%) (*p* < 0.01) (Figure 4D).

### 3.5 HXZQ regulates Nrf2/NF-κB signaling

**FIGURE 5:**
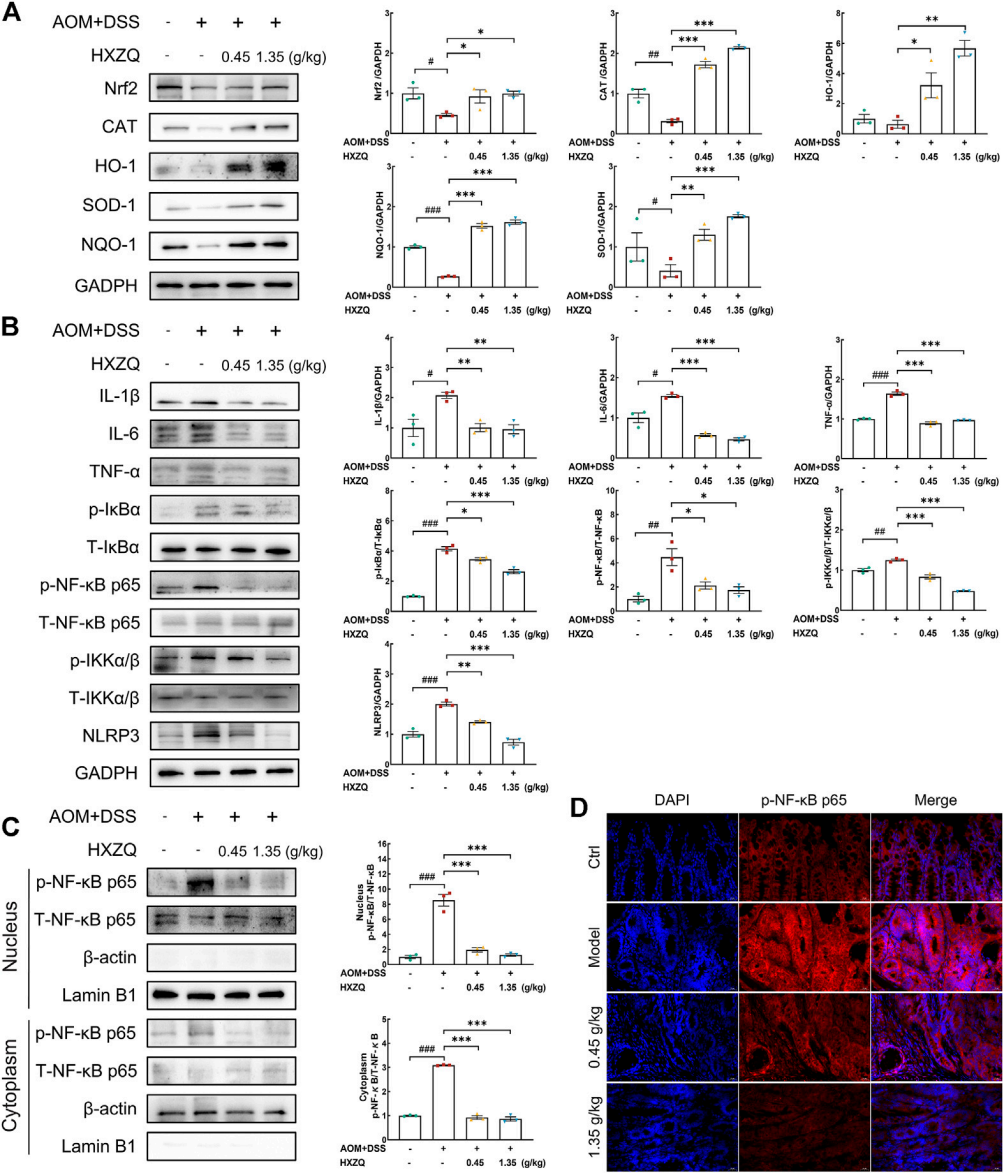
HXZQ regulated the expression of proteins in the colorectal tumors of AOM/DSS mice. **(A)** Proteins associated with oxidative stress, including Nrf2, CAT, HO-1, SOD-1, and NQO-1. **(B)** Proteins associated with inflammation, including IL-1β, IL-6, TNF-α, p/T-IκBα, p/T-NF-κB, p/T-IKKα/β, and NLRP3. **(C)** HXZQ decreased the phosphorylation level of NF-κB in the cytoplasm and inhibited its translocation from the cytoplasm to the nucleus. **(D)** Immunofluorescence for p-NF-κB was performed on colon sections (400 × ). Quantitative data were normalized by GAPDH and their corresponding total proteins and reported as fold change with respect to the expression data from the corresponding Ctrl mice (*n* = 3). Data are presented as the means ± S.E.M. and differences were compared between Ctrl and Model using Student’s t-test. Comparisons between Model, 0.45 g/kg HXZQ, and 1.35 g/kg HXZQ were performed with one-way ANOVA followed by a post hoc multiple comparisons (Dunnett) test. ^#^ *p* < 0.05, ^##^ *p* < 0.01, and ^###^ *p* < 0.001 vs. Ctrl group; *p < 0.05, **p < 0.01, and ***p < 0.001 vs. model group.

Nrf2-regulated signaling plays a crucial role in the antioxidant response and has antitumorigenic effects. Its crosstalk with NF-κB has been extensively studied. NLRP3 inflammasome, which is activated by NF-κB, is associated with CAC. HXZQ significantly upregulated the protein levels of Nrf2 (>100%) (*p* < 0.05), catalase (CAT) (>400%) (*p* < 0.001), heme oxygenase-1 (HO-1) (>400%) (*p* < 0.05), NAD(P)H quinone oxidoreductases-1 (NQO-1) (>470%) (*p* < 0.001), and superoxide dismutase-1 (SOD-1) (>220%) (*p* < 0.01) in colorectal tissues (Figure 5A). HXZQ dose-dependently downregulated the protein levels of phosphorylated inhibitor of nuclear factor kappa-B (p-IκB) (>16%) (*p* < 0.05), p-NF-κB (>52%) (*p* < 0.05), phosphorylated inhibitor of nuclear factor kappa-B kinase (p-IKK) (>33%) (*p* < 0.001), IL-1β (>51%) (*p* < 0.01), IL-6 (>63%) (*p* < 0.001), TNF-α (>40%) (*p* < 0.001), and NLRP3 (>29%) (*p* < 0.01) in colorectal tissues (Figure 5B). HXZQ inhibited the phosphorylation of NF-κB in cytoplasm while also suppressing the proportion of NF-κB translocated to the nucleus (>70%) (*p* < 0.001) (Figure 5C). The results of Immunofluorescence analysis further confirmed this result (Figure 5D).

## 4 Discussion

In the current study, HXZQ showed anti-CAC effects by improving intestinal microbiota composition, upregulating Nrf2 signaling, and downregulating NF-κB/NLRP3 signaling in AOM/DSS-induced CAC mice. An increase in the levels of pro-inflammatory cytokines and markers of oxidative stress was detected in colorectal tissues of CAC mice; in contrast, HXZQ reversed these pathological changes and increases the levels of anti-inflammatory factors. IL-10 is an anti-inflammatory cytokine, and in previous studies, *IL10*-/- mice spontaneously developed colitis, whereas upregulation of IL-10 alleviated CAC, which is consistent with our study (Zhang et al., 2016). Reducing the expression or neutralization of pro-inflammatory factors, including several types of ILs, significantly improves the symptoms of CAC, consistent with our findings (Xiao Y. T. et al., 2016; Romano et al., 2016; Menghini et al., 2019). Knockdown of *IL-21*, which is overexpressed in human colorectal cancer and CAC mice, can reverse the overexpression of IL-6 and IL-17A in CAC mice (Stolfi et al., 2011). IL-23 exacerbates inflammation and may drive the conversion of colitis to CAC by promoting the release of IL-6 and IL-17 (Yen et al., 2006; Hirano et al., 2020). ROS can induce the transition from chronic inflammation to CAC by inducing dysregulation of NF-κB signaling and stimulating the expression of pro-inflammatory cytokines such as TNF-α and IL-1 (Wang et al., 2016).

Notably, changes in pro-inflammatory cytokine levels may also affect host’s intestinal microbiota. Accordingly, IL-33-deficient mice developed a dysregulated gut microbiota (Malik et al., 2016), and altered gut microbiota in *IL-1α*-knockout mice resulted in protection against DSS-induced colitis (Nunberg et al., 2018). Disruption of homeostasis leads to changes in the intestinal microbiota and their metabolites, such as enrichment of potential pathogens and decreased butyric acid production, leading to chronic inflammation and DNA damage (Wu et al., 2013; Dzutsev et al., 2015), which are directly responsible for CAC. The ratio of Firmicutes to Bacteroidetes characterizes the function of intestinal microorganisms (Zhu et al., 2020; Wang et al., 2021). The effect of HXZQ on the intestinal microbiome of healthy adults and the gut microbiota dysbiosis mice model confirmed that HXZQ can safely improve the composition of the intestinal microbiota (Gao et al., 2022). HXZQ increased the proportion of Bacteroidetes and its affiliated beneficial bacteria and decreased the proportion of Firmicutes. Additionally, HXZQ decreased the abundance of *Candidatus_Arthromitus*, *Turicibacter*, *Dorea*, *Acinetobacter*, *Clostridium,* and *Desulfovibrio* and increased the abundance of butyric acid-producing bacteria, including *Anaerotruncus*, *Prevotella*, *Butyricimonas*, *Bacteroides*, *Rikenella*, and *Butyricicoccus* at the genus level. Based on previous research, *Dorea* tends to show a negative correlation with short-chain fatty acids (SCFA) production and a strong positive correlation with the pro-inflammatory cytokine TNF-α (Han Y. et al., 2019). *Acinetobacter* activates the NLRP3 inflammasome and consequently mediates IL-1β production (Kang et al., 2017). The growth rate of *Turicibacter* and *Desulfovibrio* is increased under inflammatory conditions (Rowan et al., 2010; Berry et al., 2015; Huang et al., 2021), and *Desulfovibrio* has been reported to induce secretion of IL-6 and IL-8 from endothelial cells (Weglarz et al., 2003).

Intestinal microbiota directly influence host metabolism (Nicholson et al., 2012). HXZQ increased L-Glutamine, glycerophosphocholine, and thiamine levels and reduced indoxyl sulfate content in CAC mice. Thiamine and glycerophosphocholine might increase the antioxidant capacity of the host. In this study, thiamine showed a significant negative correlation with Clostridium. Indoxyl sulfate was significantly negatively correlated with several butyric acid-producing bacteria as the concentration of HXZQ increased. Dietary thiamine supplementation increases the expression of Nrf2 and its downstream proteins and decreases the phosphorylation of NF-κB in goat rumen epithelial cells (Ma et al., 2021). Glycerophosphocholine is a lipid metabolite exported from cells expressing high levels of Nrf2 and is thought to prevent oxidative stress-induced cell damage (Saigusa et al., 2020; Antoine et al., 2022). L-Glutamine can inhibit the activation of NF-κB (Kim and Kim, 2017), whereas indoxyl sulfate, accumulating in the sera of mice with colon cancer, enhances the expression of pro-inflammatory cytokines (Li et al., 2015). Our data confirmed that HXZQ exerted anti-CAC effects through its anti-inflammatory properties.

Unsurprisingly, HXZQ activated Nrf2 signaling while increasing the levels of antioxidant enzymes such as CAT, HO-1, NQO-1, and SOD-1. Moreover, HXZQ inhibited the phosphorylation of IκBα, IKKα/β, and NF-κB as well as the expression of NLRP3. The accumulation of ROS during chronic inflammation leads to oxidative stress that aggravates CAC development (Wang et al., 2016). Nrf2 controls the adaptive response to oxidative stress (Hayes and Dinkova-Kostova, 2014), which can inhibit the phosphorylation of IKKα/β and IκBα, thereby inhibits NF-κB phosphorylation and nuclear translocation and thus inhibits the release of downstream pro-inflammatory cytokines (Wardyn et al., 2015). NLRP3 inflammasome is responsible for increased production of IL-1β (Iida et al., 2020), and suppressing NLRP3 inflammasome activation by inhibiting MKP1/NF-κB pathway could attenuate DSS-induced ulcerative colitis (Wei et al., 2021). HXZQ may regulate Nrf2/NF-κB signaling, thus inhibiting *NLRP3* transcription (Wang et al., 2018).

The present study has some limitations. The detailed relationship between HXZQ-mediated gut microbiota regulation and the anti-inflammatory and anti-oxidant effects that underlie its anti-CAC activity requires further investigation. Second, the main components involved in the therapeutic effect of HXZQ in the treatment of CAC need to be identified. Finally, the effects of HXZQ on other IBDs require further investigation.

Altogether, HXZQ alleviates CAC by modulating the intestinal microbiota and metabolism, activating Nrf2-mediated antioxidant response, and inhibiting NF-κB-mediated NLRP3 in mice. Our data provide a reference for the use of HXZQ as a therapeutic agent or a combination agent for clinical CAC treatment.

## Data Availability

The original contributions presented in the study are publicly available. This data can be found here: https://www.ncbi.nlm.nih.gov/sra/PRJNA860221
