# Supplementary material for: Huoxiang Zhengqi alleviates azoxymethane/dextran sulfate sodium-induced colitis-associated cancer by regulating Nrf2/NF-κB/NLRP3 signaling
Source: Front Pharmacol. 2022 Oct 21;13:1002269. doi: 10.3389/fphar.2022.1002269 (PMC9634060; doi:10.3389/fphar.2022.1002269)
Supplement: Supplementary file 1 [file DataSheet1.pdf]

Supplementary Material

Table S1 Components of HXZQ (per 1066g of the product).

Latin Name	Components
Rhizoma Atractylodis	160g
Citrus reticulata	160g
Cortex Magnoliae officilis	160g
Radix Angelicae Dahuricae	240g
Poria	240g
Pericarpium ArecaeAreca	240g
Rhizoma Pinelliae	160g
Radix Glycyrrhizae	20g
Oleum Pogostemonis	1.6ml
Oleum Folii Perillae	0.8ml

Table S2 Details of antibodies used in western blot.

Antibody	Molecular weight	Catalog number	Dilution	Company	Area
IL-1β	30 kDa	A16288	1:1000	ABclonal	Wuhan, China
IL-6	24 kDa	12912S	1:1000	Cell Signaling Technology	Beverly, MA, USA
TNF-α	25 kDa	A0277	1:1000	ABclonal	Wuhan, China
p-IκBα (Ser32/Ser36)	39 kDa	AF2002	1:1000	Affinity	Cincinnati, OH, USA
IκBα	35 kDa	ab32518	1:4000	Abcam	Cambridge, MA, USA
p-NF-κB p65 (Ser536)	65 kDa	AF2006	1:1000	Affinity	Cincinnati, OH, USA
NF-κB p65/RelA	60 kDa	A18210	1:1000	ABclonal	Wuhan, China
p-IKKα/β	87 kDa	AF3013	1:1000	Affinity	Cincinnati, OH, USA
IKKα/β	85 kDa	ab178870	1:1000	Abcam	Cambridge, MA, USA
NLRP3	100 kDa	A5652	1:1000	ABclonal	Wuhan, China
Nrf2	100 kDa	A1244	1:1000	ABclonal	Wuhan, China
CAT	60 kDa	A11220	1:1000	ABclonal	Wuhan, China
HO-1	33 kDa	A19062	1:1000	ABclonal	Wuhan, China
SOD-1	18 kDa	A12537	1:1000	ABclonal	Wuhan, China
NQO1	31 kDa	A19586	1:1000	ABclonal	Wuhan, China
GAPDH	37 kDa	E-AB-20032	1:2000	Elabscience	Wuhan, China
Lamin B1	70 kDa	A11495	1:1000	ABclonal	Wuhan, China
β-Actin	43 kDa	sc-47778	1:1000	Santa Cruz	Dallas, TX, USA
p-NF-κB p65	65 kDa	GB13025-1	1:1500	servicebio	Wuhan, China
Goat Anti-Rabbit (H+L)	/	E-AB-1003	1:4000	Elabscience	Wuhan, China
Goat Anti-Mouse (H+L)	/	E-AB-1001	1:4000	Elabscience	Wuhan, China

Cy3 conjugated Goat Anti-mouse IgG (H+L)	/	GB21301	1:600	servicebio	Wuhan, China

Table S3 Relative abundance of top 50 genera.

Sample	Mean of Ctrl	Mean of Model	Mean of 0.45g/kg	Mean of 1.35g/kg
Lactobacillus	0.2534	0.0749	0.0493	0.0273
Oscillospira	0.0122	0.0348	0.0409	0.0354
Allobaculum	0.0672	0.0149	0.0222	0.0080
Coprococcus	0.0096	0.0174	0.0114	0.0176
Phascolarctobacterium	0.0134	0.0090	0.0188	0.0141
Desulfovibrio	0.0108	0.0129	0.0135	0.0071
Ruminococcus	0.0034	0.0102	0.0176	0.0119
Clostridium	0.0029	0.0135	0.0113	0.0022
Bacteroides	0.0037	0.0049	0.0067	0.0100
Sutterella	0.0038	0.0032	0.0087	0.0058
[Ruminococcus]	0.0050	0.0079	0.0031	0.0033
Paraprevotella	0.0011	0.0019	0.0046	0.0059
Turicibacter	0.0003	0.0048	0.0027	0.0025
Adlercreutzia	0.0035	0.0027	0.0021	0.0012
Akkermansia	0.0020	0.0010	0.0024	0.0028
Mucispirillum	0.0013	0.0013	0.0029	0.0026
Parabacteroides	0.000854	0.000956	0.00260	0.00371
Flexispira	0.00161	0.00147	0.00239	0.00221
Bifidobacterium	0.00197	0.00183	0.00177	0.00045
Prevotella	0.00046	0.00100	0.00103	0.00253
Alistipes	0.000861	0.000758	0.00141	0.00151
Dehalobacterium	0.000598	0.000728	0.00169	0.00139
Roseburia	0.00180	0.000393	0.000213	0.000402
Anaerotruncus	0.000123	0.000501	0.00104	0.000343
Odoribacter	0.000597	0.000438	0.000322	0.000333
Helicobacter	0.000332	0.000228	0.000350	0.000763
Rikenella	0.000702	0.000143	0.000448	0.000344
Coprobacillus	0.000961	0.0000990	0.000162	0.000284
Butyricicoccus	0.0000916	0.000142	0.000559	0.000349
Dorea	0.0000859	0.000561	0.0000661	0.000368

Faecalibacterium	0.000295	0.000123	0.000242	0.000203
Blautia	0.000258	0.0000898	0.000291	0.000131
Shigella	0.0000558	0.000230	0.000163	0.000313
Candidatus_Arthromitus	0.000146	0.000284	0.000123	0.000177
Anaeroplasma	0.0000109	0.000120	0.000168	0.000392
Streptococcus	0.000190	0.000135	0.000160	0.000176
Acinetobacter	0.000163	0.000193	0.000152	0.000111
Sphingomonas	0.0000413	0	0.000240	0.000225
HB2-32-21	0.000268	0.000211	0	0
AF12	0.0000394	0.0000898	0.000215	0.000116
Psychrobacter	0	0.000064	0.000177	0.000156
Anaerostipes	0.000392	0	0	0
p-75-a5	0.000187	0.0000671	0.0000237	0.0000717
Rhodanobacter	0	0.0000754	0.0000496	0.000163
Olsenella	0.000195	0.0000244	0	0
Pediococcus	0.0000419	0.0000195	0.0000755	0.0000815
Bacillus	0	0	0	0.000208
Burkholderia	0	0.0000377	0.0000779	0.0000840
Selenomonas	0.0000832	0.0000479	0.0000332	0.0000147
Butyricimonas	0	0.00000471	0	0.000146

Table S4 Significantly different metabolites.

Adduct	Name	Mean of Ctrl	Mean of Model	Mean of 0.45g/kg	Mean of 1.35g/kg
(M-H)-	7Z, 10Z, 13Z, 16Z, 19Z-Docosapentaenoic acid	270524.71	282791.97	218731.58	185470.62
(M-H)-	Taurocholate	127181.60	58754.67	126807.41	26679.64
(M-H)-	Linoleic acid	1519141.61	1591996.18	1345549.73	1302324.97
(M-H)-	Nervonic acid	20221.28	24767.04	19184.19	14802.48
(M-H)-	Dihomo-gamma-Linolenic Acid	177304.03	170786.09	137083.96	104893.33
(M-H)-	Indoxyl sulfate	1939414.27	2552495.92	2062692.58	921513.70
(M-H)-	(4Z,7Z,10Z,13Z,16Z,19Z)-4,7,10,13,1 6,19-Docosahexaenoic acid	1521105.78	1369352.28	1107882.89	1055520.35
(M-H2O-H)-	Glycerol 3-phosphate	97262.27	70241.97	55263.81	50144.70
(M-H)-	13(S)-HODE	62123.35	75383.74	64150.90	55630.40
(M+Na-2H)-	1-Oleoyl-L-alpha-lysophosphatidic acid	40262.97	42804.42	33272.34	23343.57
(M-H)-	Thymidine	9207.25	22668.88	14565.43	11546.58
(M-H)-	cis-9-Palmitoleic acid	536554.92	569130.52	534192.88	384839.89
(M-H)-	3-Indolepropionic acid	18248.48	8677.56	10237.89	6650.87
(M-H)-	Cysteine-S-sulfate	13026.66	7988.84	8100.35	4586.04
(M-H)-	Methylmalonic acid	23343.53	6182.25	12728.54	9644.63
(M-H)-	Hydroxyisocaproic acid	15749.16	36659.31	29615.85	21249.33
(M+H)+	Cytidine	20766.87	10262.96	21429.67	17606.45
(M+H-H2O)+	Erucic acid	20411.72	27731.82	15393.59	19506.92
(M+H)+	L-Glutamine	103719.53	48016.93	109844.86	68615.63
(M+H)+	Erucamide	891602.42	1057821.56	658067.55	859032.96
M+	Glycerophosphocholine	18683.76	15109.77	15788.03	36966.83
(M+H)+	1-Palmitoyl-2-hydroxy-sn-glycero-3-phosphoethanolamine	17833.47	16146.20	18409.23	22066.30
(M+NH4)+	L-Pipecolic acid	16731.06	19643.25	17448.34	13493.46
(M+H-H2O)+	7-Oxocholesterol	10093.45	11522.08	11347.20	16496.49
(M+H)+	DL-Norvaline	78648.58	63752.40	74209.15	75306.24
(M+H-H2O)+	DL-2-Aminoadipic acid	9281.73	8366.35	12520.35	12593.55
(M+H)+	Pantothenate	41683.67	39708.93	46970.19	46123.24
(M+CH3COO+2H)+	Cyclohexylamine	20736.15	7842.94	5827.07	9707.57
M+	Thiamine	10481.51	3631.43	4542.13	8331.41

Figure List

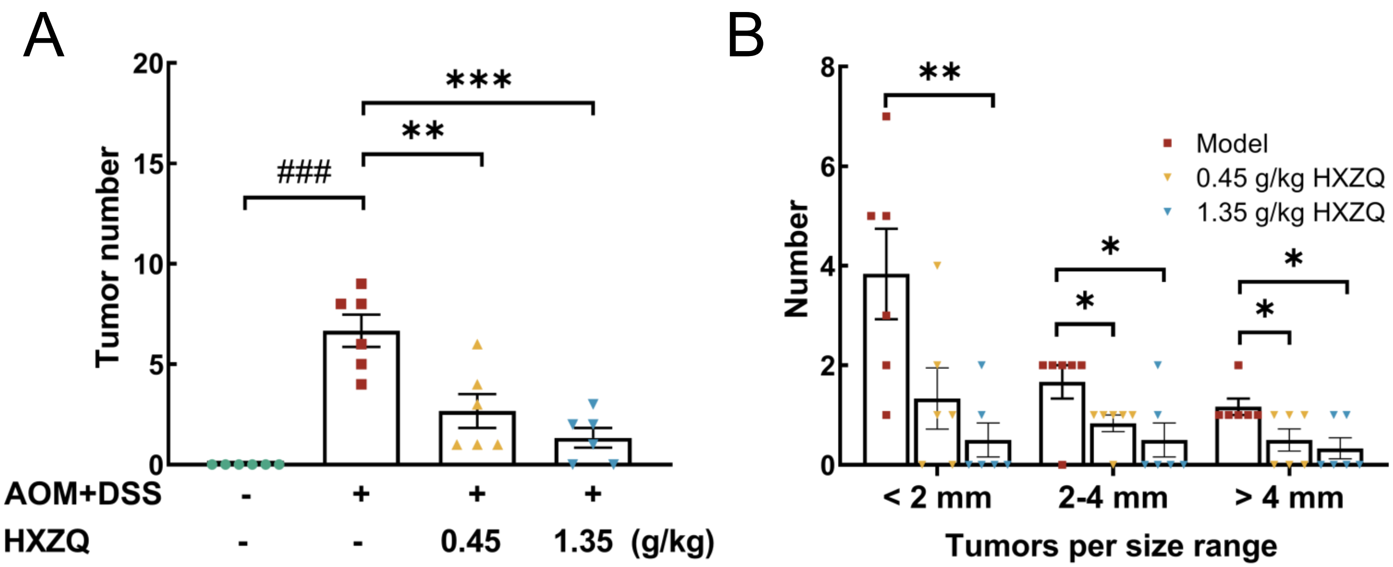

Figure S1 Number (A) and size (B) of colorectal tumors. Data are presented as the means ± S.E.M.. Differences were compared between Ctrl and Model using Student's t-test, and comparisons between Model, 0.45 g/kg HXZQ, and 1.35 g/kg HXZQ were performed with one-way ANOVA followed by a post hoc multiple comparisons (Dunnett) test. $^{\#\#\#}p < 0.001$ *vs.* Ctrl group; $^{*}p < 0.05$, $^{**}p < 0.01$, and $^{***}p < 0.001$ *vs.* model group.

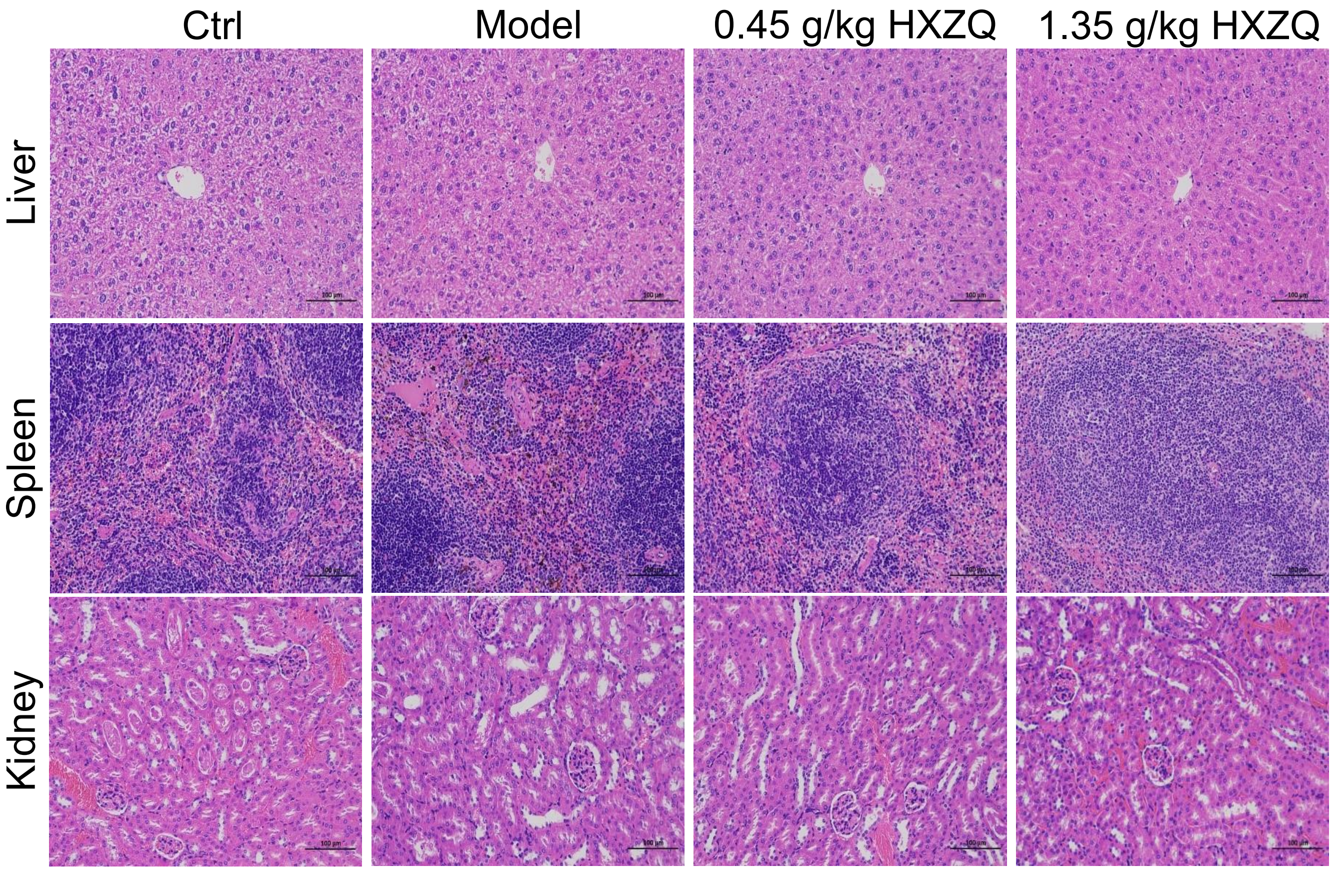

Figure S2 Histopathological analysis of the liver, spleen, and kidney via H&E staining (200×; scale bar: 100 μm).

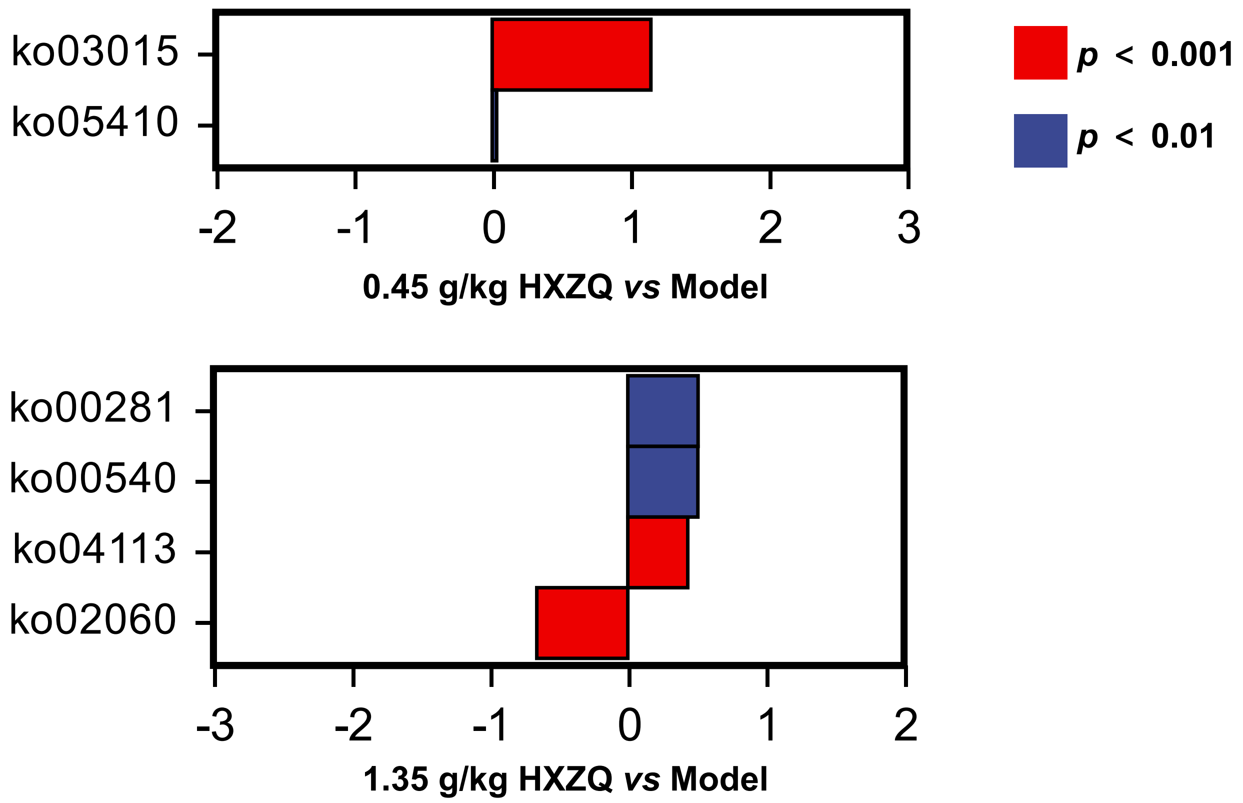

Figure S3 Discriminative functional pathway abundant between HXZQ (0.45 g/kg)-treated group vs. model group, and HXZQ (1.35 g/kg)-treated group vs. model group.
